# How the Toxin got its Toxicity

**DOI:** 10.3389/fphar.2020.574925

**Published:** 2020-12-14

**Authors:** Timothy N. W. Jackson, Ivan Koludarov

**Affiliations:** ^1^ Australian Venom Research Unit, Department of Pharmacology and Therapeutics, University of Melbourne, Melbourne, Australia; ^2^ Animal Venomics Group, Justus Leibig University, Giessen, Germany

**Keywords:** toxin, venom, evolution, molecule, function, genomics, duplication, gene expression

## Abstract

Venom systems are functional and ecological traits, typically used by one organism to subdue or deter another. A predominant subset of their constituent molecules—“toxins”—share this ecological function and are therefore molecules that mediate interactions between organisms. Such molecules have been referred to as “exochemicals.” There has been debate within the field of toxinology concerning the evolutionary pathways leading to the “recruitment” of a gene product for a toxic role within venom. We review these discussions and the evidence interpreted in support of alternate pathways, along with many of the most popular models describing the origin of novel molecular functions in general. We note that such functions may arise with or without gene duplication occurring and are often the consequence of a gene product encountering a novel “environment,” i.e., a range of novel partners for molecular interaction. After stressing the distinction between “activity” and “function,” we describe in detail the results of a recent study which reconstructed the evolutionary history of a multigene family that has been recruited as a toxin and argue that these results indicate that a pluralistic approach to understanding the origin of novel functions is advantageous. This leads us to recommend that an expansive approach be taken to the definition of “neofunctionalization”—simply the origins of a novel molecular function by any process—and “recruitment”—the “weaponization” of a molecule via the acquisition of a toxic function in venom, by any process. Recruitment does not occur at the molecular level or even at the level of gene expression, but only when a confluence of factors results in the ecological deployment of a physiologically active molecule as a toxin. Subsequent to recruitment, the evolutionary regime of a gene family may shift into a more dynamic form of “birth-and-death.” Thus, recruitment leads to a form of “downwards causation,” in which a change at the ecological level at which whole organisms interact leads to a change in patterns of evolution at the genomic level.

## Introduction

### Venom Systems are Functional Ecological Traits

Venom systems are functional and ecological traits, used by one organism to subdue, deter, or surreptitiously feed upon, another (Jackson et al., 2019). A venom system is composed of a secretion—the venom itself—the tissue that produces that secretion (e.g., a “venom gland”), and a delivery mechanism (e.g., fangs or a stinger) that inoculates the secretion to the target organism. The functional constituents of the venom itself are physiologically active molecules, largely proteins and peptides, known as “toxins.” As these toxins have been designed (by natural selection) to function outside the body of the producing organism, they have been referred to as “exochemicals,” and their function has been described as “exophysiological.” That exochemicals are related to endophysiological counterparts has long been understood, but the evolutionary pathways through which a typical enzyme or peptide with a regulatory function within the body of the producing organism becomes a weaponised, exophysiological “toxin,” remain only partially understood.

### Recruitment vs. Restriction

The dominant view in evolutionary toxinology has reflected the dominant view in molecular evolution more broadly. What is referred to as “recruitment” in toxinology is a coarse-grained description of the acquisition of a novel function at the molecular level that mirrors Ohno’s influential “neofunctionalization (NF)” model, in which “random” (unselected) gene duplication results in a functionally redundant copy or copies (Ohno, 1970). This redundancy relaxes constraints on the gene network by rendering the accumulation of mutations by any one copy functionally neutral, thereby facilitating exploration of sequence space. When this random walk results in the discovery of a “good trick,” positive selection fixes the mutation and NF has occurred. In the toxinological literature, this has been described as the duplication of a gene with a “physiological” function conferring the possibility of “recruitment to the venom gland” and “weaponization”—the acquisition of a toxic function within venom (e.g., Fry and Wüster, 2004; Lynch, 2007). Though this model has been widely accepted in toxinology for much of the twenty first century, it has also been criticised on the basis of a lack of experimental evidence. In 2014, Hargreaves et al. suggested that subfunctionalisation, in which duplication of a multifunctional parent gene enables the segregation of functions among daughter genes, is a more likely process for the origins of toxin genes (Hargreaves et al., 2014). They termed this process “restriction” (rather than recruitment), suggesting that following the acquisition of a novel, toxic function, a gene which was previously widely expressed would have its expression restricted to the venom gland (to avoid auto-toxicity), rather than recruited to it.

In order to compare and contrast “restriction” and “recruitment,” as well as other models of the acquisition of toxic function, it is necessary to both review the diversity of models that have been proposed within the molecular evolution literature, and to consider what sort of evidence would be required to differentiate them empirically. In this article, we briefly review these models and then consider their applicability to toxin evolution based on evidence from a number of studies. We also consider the appropriate definition of “function” in a biological context and distinguish the origins of a novel function from the origins of mere activities. We then discuss in detail data arising from our reconstruction of the evolutionary history of the multigene family phospholipase a2g2, which is a major component of the venoms of viperid snakes. We consider the applicability of a range of gene evolution models to this data. Finally, we suggest that the terms “NF” and “recruitment” be considered general terms—the former for the origins of novel molecular functions and the latter for the “weaponization” of molecules as toxins in venom systems, regardless of the precise sequence of events that facilitated the origin of this novel toxic function.

## Review of Models

### Ohno’s Dilemma; Neofunctionalization, Subfunctionalization and Moonlighting

Despite the continued influence of Ohno’s model, a vibrant literature on gene duplication has subsequently produced many other models, a number of which are reviewed in (Innan and Kondrashov, 2010), which either expand upon or contradict the basic NF framework. A number of these attempt to account for what has been dubbed “Ohno’s Dilemma” (Bergthorsson et al., 2007)—how do duplicate genes survive long enough under neutral conditions to acquire the necessary changes of sequence or expression regulation that result in functional divergence? Several possible fates for duplicates are frequently discussed. One likely outcome is that duplicates are pseudogenised, either by further random events or as a direct result of selection stabilizing gene dosage (Bergthorsson et al., 2007; Birchler and Veitia, 2012).

**FIGURE 1:**
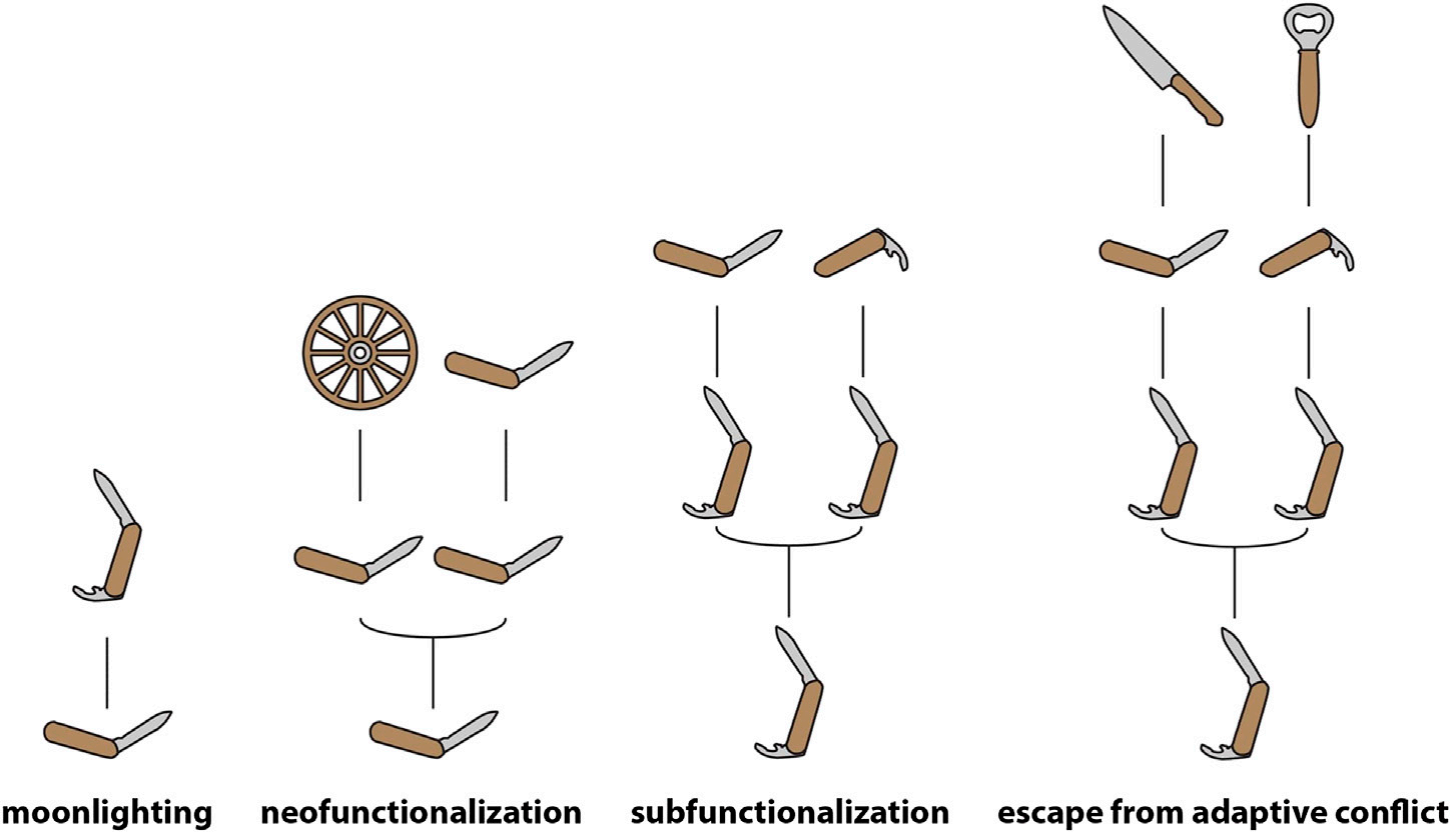
Models of gene evolution. Schematic representation of the most prominent models of gene evolution, with genes and their functions being represented as human tools. Note that moonlighting occurs in the absence of any duplication, with the product of a single gene fulfilling multiple functional roles—pleiotropy of this kind is likely extremely widespread (Paaby and Rockman, 2013), and though genes are depicted as having single functions at the base of the moonlighting and neofunctionalization models in this figure, this should be understood as purely illustrative.

Two models are most widely invoked to describe the fate of duplicates that survive and go on to fulfil functional roles—“subfunctionalization” (SF) and “NF” (Force et al., 1999; Conant and Wolfe, 2008; see Figure 1 for illustrations of common models of gene evolution). In the former the parent gene performed multiple functions, which are subsequently distributed between the duplicates, each of which may acquire function-impairing “degenerate” mutations resulting in the necessary maintenance of both copies to fulfil the functional role of the parent gene. In the latter, a novel function is discovered during the period of relaxed constraint immediately following duplication.

Both NF and SF give pride-of-place to gene duplication as a facilitator of functional change and thus it may appear as though duplication must precede the origin of novel functions. However, it should also be noted that novel functions may emerge as the result of changes of tissue-specific expression patterns in the absence of duplication, a process known as “gene sharing” (Wistow and Piatigorsky, 1987) or “moonlighting” (Copley, 2014). Following this period of functional sharing, duplication may facilitate the emergence of distinct proteins capable of subdividing the shared function between them (Force et al., 1999) and specializing for one of the ancestral functions (Hughes, 1994). Even in SF, which does not directly describe the origins of a “novel function” (since the gene in question is already pleiotropic during the period described by the model), the secondary function is “novel” (i.e., originates later in the evolutionary history of the gene) relative to the “original” function. Thus, in these scenarios acquisition of a novel function occurs prior to duplication (Piatigorsky and Wistow, 1991). It should be noted, however, that most models to do not appear to take into account the role that widespread pleiotropy and redundancy (which results in many mutations being functionally neutral) must play in the origins of novel molecular functions (Wang et al., 2010). These processes, which are intimately involved in the evolution of complexity, must surely complicate analyses of the emergence of such functions. Toxin genes may represent a special case here, in that their toxicity may prevent them from fulfilling multiple functional roles in a phenotype (however, see Casewell et al., 2012). Detailed discussion of these factors is beyond the scope of the present article, but their potential importance should be kept in mind when models are discussed.

### Dosage Balance

The “classic” models have been further nuanced by the recognition of distinct forms of SF and NF. “Escape from adaptive conflict” (EAC), in which a novel specialized function emerges following the partitioning of the ancestral function (Innan and Kondrashov, 2010), may be considered an extension of “SF,” though the difference between EAC and “specialization,” which was described by Hughes (1994), *prior* to Force et al. (1999) proposal of SF, appears minimal. The NF paradigm, on the other hand, has been extended by models in which duplication is intrinsically advantageous and thus may be positively selected. In these latter models, the initial benefit of duplication may be the consequence of increased gene dosage, increased robustness (protection against deleterious point mutations), or the “spontaneous origin” of a novel “function” (though “activity” or “propensity” would be more appropriate terms for such spontaneous novelties, cf. Innan and Kondrashov, 2010; see below for further discussion). In all such cases, the consequent accumulation of redundant copies in gene family networks may generate a hotspot for functional novelty. As recognized by Ohno (1970) and supported by much subsequent research, gene duplication often results in an increase in the dosage of the product encoded by the multiplied genes (e.g., Conant et al., 2014; Margres et al., 2017). In light of this, much of the recent literature on gene duplication centers on the importance of gene dosage in determining the fate of duplicates. A key observation in this regard is the divergent fates of duplicates that originate in whole genome duplication (WGD) events and those that are locally (segmentally) duplicated (LD) (Birchler and Veitia, 2012; Conant et al., 2014). In the case of WGD, preserved duplicates are typically those with numerous interaction partners with which they must maintain precise stoichiometric balance—if one half of a pair is lost, a dosage imbalance may occur. Conversely, duplicates preserved after LD tend to be genes with few interaction partners—they can persist in the genome because their origin does not cause a dosage imbalance. Such genes are “dosage-insensitive.” Expression levels of many genes may vary considerably within a population—while heritable, much of this variation is stochastic and may be neutral or nearly neutral. On the other hand, it may be a cryptic contributor to diseases (Cheung et al., 2003) and also represents some of the “standing variation” which may facilitate the evolution of complex traits, including venoms (see below).

### Selection for Increased Dosage

**FIGURE 2:**
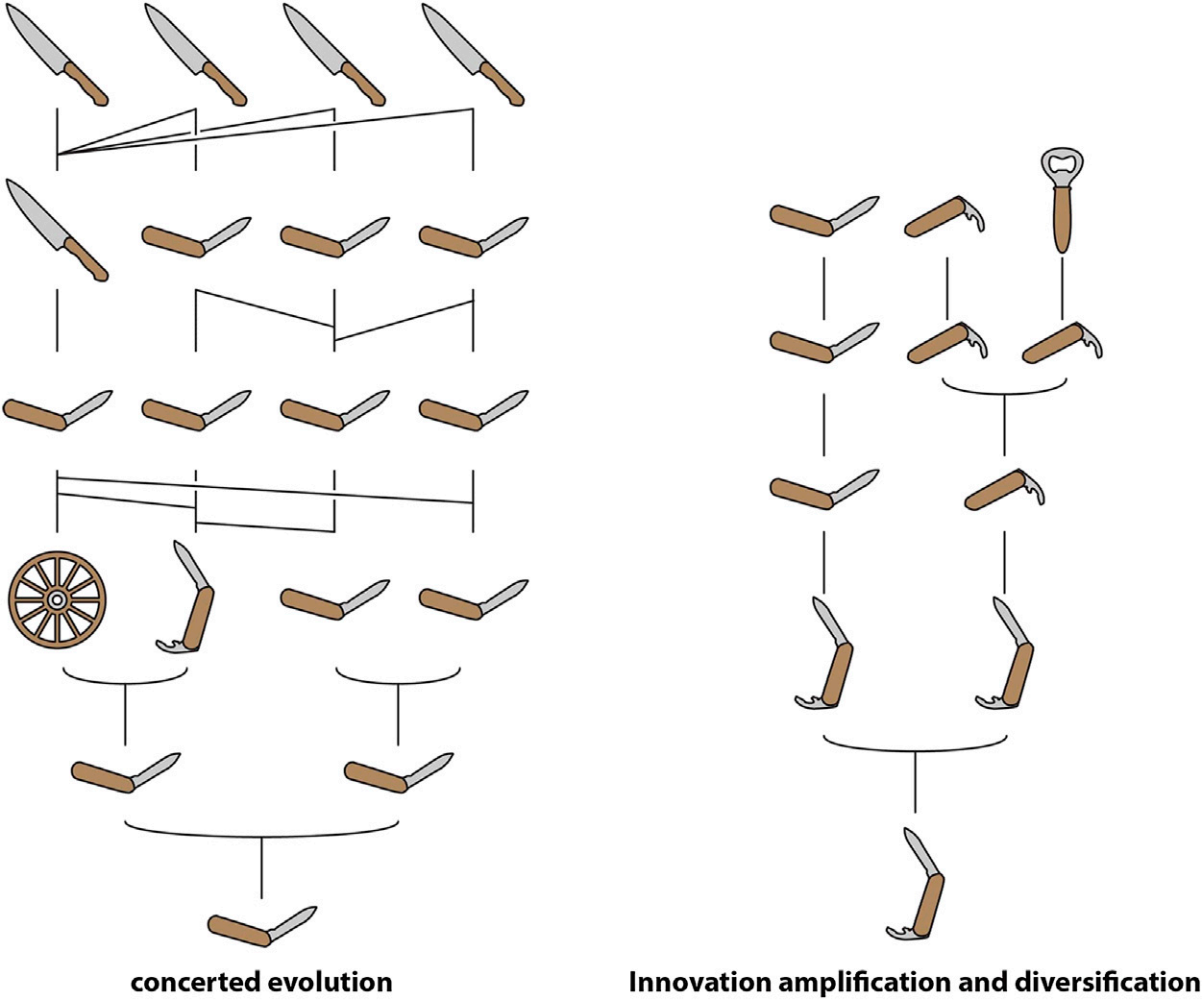
Schematic representation of “concerted evolution” and “innovation-amplification-diversification” using human tools. Note that gene conversion/unequal crossover in concerted evolution result in each gene within a homogenized tandem array having multiple ancestors in the previous generation.

In certain circumstances, an increase in gene dosage may be directly selected for, a possibility highlighted by models such as “concerted evolution” (CE) and “innovation-amplification-diversification” (IAD) (Figure 2). Since the 1970s, CE has been a popular model for explaining the evolution of multigene families with members that share highly similar sequences in regions that encode the mature product (Nei and Rooney, 2005). In this model, mutations acquired by one member are either shared with other members or reversed, due to gene conversion or unequal crossing over among all members of the family. Thus, an array of highly similar genes is maintained within the genome of a single species, facilitating the expression of a large quantity of the product of these genes. The canonical example of CE is ribosomal RNA. The model was originally proposed to explain the curious fact that the ribosomal RNA genes of *Xenopus laevis* and *X. muelleri* exist in tandem arrays of as many as 450 copies which differ very little within each species but diverge by up to 10% between the two (Brown et al., 1972).

IAD is a model that combines elements of CE with the SF and NF model. In this model, the parent gene possesses a “weak secondary activity” (as in SF) that results in selection for increased gene dosage (Näsvall et al., 2012). This selection pressure either drives gene duplication or results in the preservation of “random” duplicates, resulting in a redundant array that enables specialisation for either the parent or secondary function, or the origin of entirely novel functions (as in NF). Pluralistic models like IAD have become increasingly popular as the combination of genomic sequences and gene expression data has revealed the complexity involved in the evolution of novel functions.

### Layers of Evidence; Activity vs. Function

Indeed, differentiating between the various models described above requires multiple layers of evidence. It is not enough to show that duplication has occurred in a gene family and associate it with the diverse functions within that family, it is necessary to pinpoint the timing of the duplication events in relation to the origins of the novel functions. Without knowing whether a function emerged prior or subsequent to duplication, for example, it is impossible to differentiate between SF and NF. Furthermore, reconstructing the evolutionary history of gene expression patterns is necessary. This is because novel functions may emerge as the result of changes in expression and thus the “environment” of the gene product, which is composed of the other gene products available to interact with (Jackson et al., 2019). Such a shift in environment may be more significant in some cases than a change at the level of gene sequence. This point is particularly relevant for the acquisition of a novel toxic function (e.g., a role in venom) because toxins are “exochemical,” meaning that they find their interaction partners (targets) in the bodies of other organisms.

It is also important to stress the distinction between activity and function. A change in gene sequence or even expression site may confer a novel activity to a gene product, but in biology a novel function should only be recognised when that shift makes a contribution to the ecology of the organism (Jackson and Fry, 2016). A change in activity detectable in the laboratory may therefore be little more than an epiphenomenal property of a gene product, and not a true function. This is once again of particular relevance when considering the origins of toxic functions. As discussed above, venom is a functional and intrinsically ecological trait. Toxins, as the active components of venoms, possess an equally ecological function—they are, along with other components of an organism’s “exophysiology” (e.g., pheromones), molecules that mediate interactions between organisms. We recognise that it may be exceptionally difficult to experimentally demonstrate a functional role for a putative “toxin” in the ecology of a venomous animal; however, we make the aforementioned distinction between activity and function due to its theoretical significance in evolutionary biology. When “layers of evidence” are combined—from *in vitro* activities to the presence of specialised anatomical structures for venom delivery to observations consistent with the ecological deployment of venom—it may be entirely reasonable to infer the functional role of a given gene product.

## Evidence for Gene Evolution models Within Toxinology

### Redundancy and “Birth-and-Death”

The idea that redundancy facilitates the evolution of novelty did not originate with Ohno but, in the context of biological evolution, goes back at least to Darwin, who noted that:

“…two distinct organs, or the same organ under two very different forms, may simultaneously perform in the same individual the same function, and this is an extremely important means of transition….” (Darwin, 1859)

**FIGURE 3:**
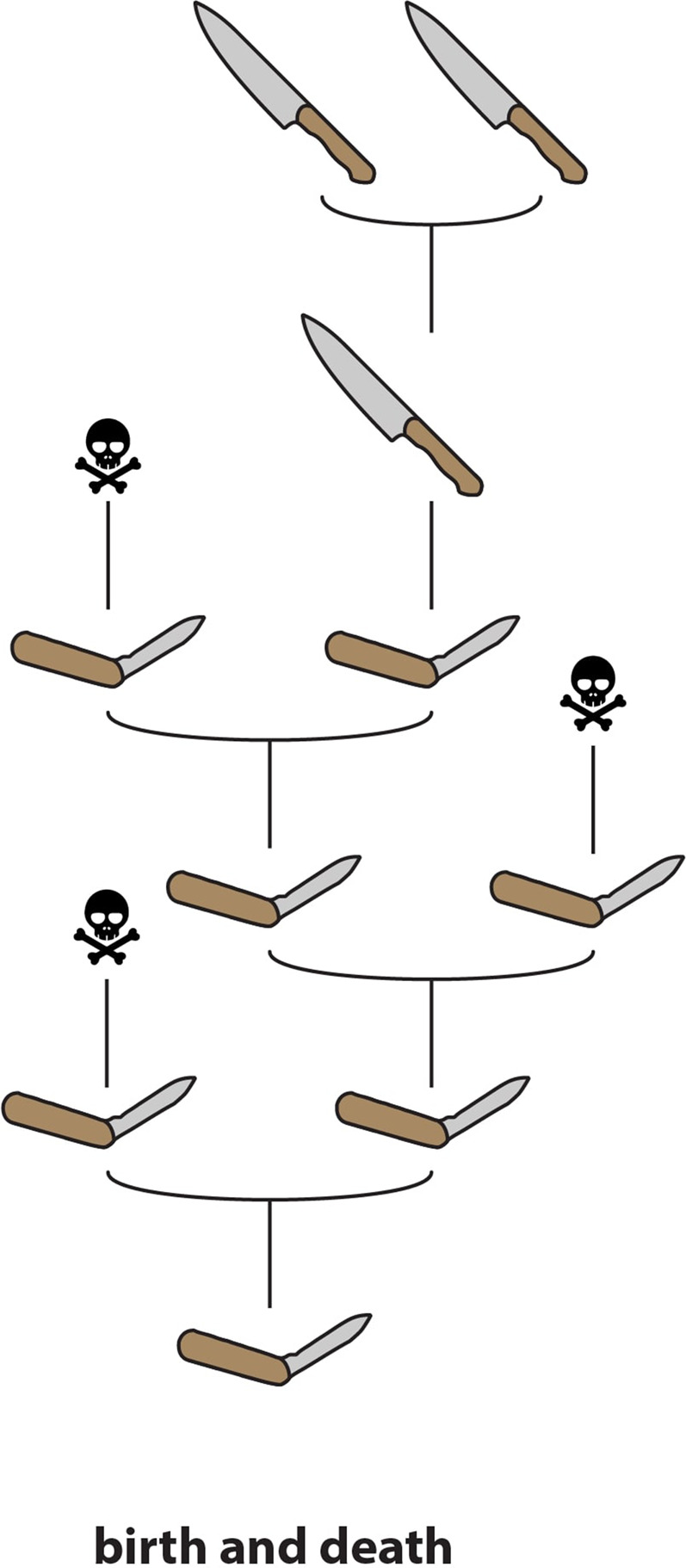
Schematic representation of “birth-and-death” molecular evolution. Genes are continuously duplicated; some go on to develop novel functions whereas others are pseudogenized (“death”).

The fact that duplication and redundancy are central to the evolution of novel functions within toxin multigene families has long been recognised (Nakashima et al., 1993; Duda and Palumbi, 1999) and is supported by a considerable body of evidence. However, it is one thing to note that toxins are typically members of multigene families, and that redundancy within these tandem arrays facilitates the evolution of new or more specialised toxic activities (i.e., to describe the evolution of these gene families as conforming to a process of “birth-and-death”—Nei and Rooney, 2005; Figure 3), and another thing to assert that “NF” (in the sense of Ohno, 1970) is involved in the *initial acquisition of a functional role in venom*. Garnering evidence in support of this latter assertion is considerably more challenging.

The inference that toxin genes are “recruited” from non-toxin endophysiological precursors (often referred to simply as “body” genes) has been drawn from the fact that toxin genes are typically members of widely expressed gene families. In a seminal study that is frequently referenced to support the assertion that toxin genes are “recruited” following duplication of genes encoding “body proteins,” information concerning the expression patterns and functions of homologues of toxin genes was compiled from the literature (Fry, 2005). Another study widely cited in support of the “recruitment hypothesis” (Lynch, 2007) references an earlier study demonstrating that the type-1 phospholipase a2 genes that encode toxins in the venoms of snakes in the family Elapidae are closely related to genes primarily expressed in the pancreas (Fujimi et al., 2002). In both Fry (2005) and Lynch (2007), the placement of toxin genes within families that include non-toxin homologues, along with the fact that “birth-and-death” is occurring within these families, is used to support the conjecture that gene duplication is required for toxin recruitment, however, no specific evidence in support of this hypothesis is provided.

Among the best evidence for the classic recruitment hypothesis is provided in a series of studies investigating the acquisition of a novel function in the venom of Australian elapid snakes by the activated form of the coagulation factor X (fXa) (Reza et al., 2005; Reza et al., 2006; Kwong et al., 2009). These studies identified multiple copies of the gene in two species of elapid, with different tissue-specific sites of expression. In *Tropidechis carinatus*, the ancestral coagulation factor-encoding gene was expressed in the liver, whereas a derived form, with modifications enhancing its toxic function, was expressed in the venom gland (Reza et al., 2005; Reza et al., 2007). Three distinct forms were sequenced for *Pseudonaja textilis*, two expressed in the liver and one in the venom gland. One of the liver-expressed forms was expressed at extremely low levels and was structurally intermediate between the ancestral gene (encoding the coagulation factor) and the derived toxin form (Reza et al., 2006). This was interpreted as evidence of duplication of the gene expressed in the liver, with subsequent recruitment to the venom gland via mutations in the regulatory regions of the genes, which were described in subsequent papers (Reza et al., 2006; Kwong et al., 2009). Another example of “NF” (*sensu* Ohno) in a venom system is the recruitment of a specialised form of insulin as a toxin in the venom of the piscivorous cone snails *Conus geographus* and *C. tulipa* (Safavi-Hemami et al., 2015). These weaponised insulins are exhibit a structure that is convergent with vertebrate insulins (and thus divergent from the endophysiological mollusc insulin they are descended from) and remarkably similar to the endogenous insulins of fish. This example furnishes evidence not only of NF in the evolution of a novel, toxic form of a protein species, but also of the taxon-specific targeting of the toxin, making it a striking example of the opportunity presented by venom systems as models linking molecular evolutionary pathways to ecology.

As mentioned previously, Hargreaves et al. (2014) took exception to the general acceptance of the “recruitment hypothesis” in the toxinological literature despite lack of widespread evidence in support of it. Hargreaves et al. used the fact that toxin gene homologues are often widely expressed in various bodily tissues, including in the oral glands, to argue for an alternate model—termed “restriction”—in which widely expressed genes are restricted to the venom gland after acquiring a toxic function. They further suggested that restriction should be considered a form of SF, rather than NF.

### No “One Size Fits All” Model of Toxin Evolution

In fact, many different models have been applied to explain observed patterns of toxin evolution, suggesting that there is no “one size fits all” model. “Birth-and-death” does seem to be a very common process in toxin multigene families, but CE in which all members of a tandem array of toxins possess identical or near-identical sequences (e.g., Moran et al., 2008) has also been observed. Indeed, both processes may occur in the same system simultaneously, as recently demonstrated within the toxin gene family Nv1 of the anemone *Nematostella vectensis* (Sachkova et al., 2019). Contrary to the reasonable intuition that a toxic gene product must be specialised for delivery to other organisms due to the risk of auto-toxicity, moonlighting may also be relatively common, and examples have been described in platypus (Wong and Belov, 2012), parasitoid wasp (Martinson et al., 2017) and snake (Vonk et al., 2013) venom systems. Unlike “birth-and-death” and CE, evidence of moonlighting is directly relevant to the origins of a toxic function within a gene family.

More recently, multiple studies have provided evidence that selection for increased dosage may play an important role in the evolution of toxin gene families and the accumulation of duplicate genes which subsequently form redundant arrays which enable the accumulation of mutations and thus functional diversification (Margres et al., 2017; Sachkova et al., 2019; Giorgianni et al., 2020) At least one of these studies has explicitly interpreted their evidence as an example of “IAD,” although they highlight the similarity of this model with “EAC” and suggest that either of these models may account for the pattern they observe (Giorgianni et al., 2020). This same study also interpreted their evidence as support of the “recruitment hypothesis,” because the non-toxin gene most closely related to those that encode toxins is not expressed in the venom gland. However, it should be noted that both IAD and EAC posit an ancestral gene with (at least) dual functions (Innan and Kondrashov, 2010; Näsvall et al., 2012). If these models explain the origins of a toxic function, it is necessary that that function be one of the ancestral functions, i.e., that it is present *prior* to duplication. This seems to contradict the spirit of the “recruitment hypothesis” which states that toxin genes are recruited into the venom arsenal (i.e., acquire their toxic function) subsequent to the duplication of a gene encoding a “bodily protein.”

### Genomic Data Provide Additional Insight

The use of genomic sequences has enabled reconstruction of the evolutionary history of gene families at an unprecedented level of detail. Unsurprisingly, these data have shed light on the molecular evolutionary processes involved in the origins of toxin genes. Analysis of the genome of the non-venomous Burmese python (*Python bivittatus*) indicated that venom gene homologues are expressed in a wide variety of tissues, including in many cases the rictal gland (Reyes-Velasco et al., 2014). The rictal gland is part of the same dental/labial gland complex as the venom gland (Jackson et al., 2017) and in species of snake that possess both a venom gland and a rictal gland (often only one or the other is present), the two glands exhibit markedly similar gene expression profiles (Fry et al., 2013). Given the complexity involved in deconvoluting the plethora of transitional forms of oral gland and “venom system” development in snakes, it is impossible to say whether or not the common ancestor of all snakes was likely “venomous”—i.e., used its toxic oral secretions in prey subjugation (Jackson et al., 2017).

Regardless, expression of toxin homologues (and indeed toxic proteins) in oral glands is widespread in non-venomous vertebrates, for example leopard geckos (Hargreaves et al., 2014) and mice (Hiramatsu et al., 1980). Reyes-Velasco et al. (2014) used this evidence from the python genome to formulate their “stepwise intermediate nearly neutral evolutionary recruitment” (SINNER) model of venom evolution, in which toxin precursor genes which are constitutively expressed at low levels in the oral glands have their expression elevated specifically in the oral gland (presumably via selection for a toxic function, though this is not specified), and then reduced in other tissues to avoid auto-toxicity (a process presumably selected for following specialisation for the toxic function). The model does not explicitly invoke a role for gene duplication in the origin of the novel function, but it is suggested that duplication would remove constraints and thus facilitate specialisation for a toxic function. The model is therefore very similar to IAD and EAC, in which duplication following a period of “gene sharing” (i.e., moonlighting) enables increased gene expression (in IAD) and specialisation. SINNER is therefore another name for a sequence of events which already has several very similar formulations – “specialisation,” EAC, and IAD (Hughes, 1994; Innan and Kondrashov, 2010; Näsvall et al., 2012).

**FIGURE 4:**
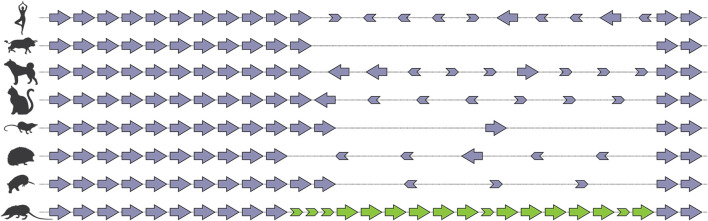
Schematic representation of tissue kallikrein gene cluster in selected species of mammals after (Casewell et al., 2019). *Solenodon* venom genes are colored green, “physiological” genes are colored violet. Note that all expansion occurs within the same region of the cluster, and that much “exonic debris” remains after the partial deletion of genes, a characteristic sign of “birth-and-death”

There are many examples, from across the animal kingdom, of toxin genes which have been recruited from multigene families (Fry et al., 2009). This suggests that an ancestral propensity to duplicate may be a property that “exapts” (Gould and Vrba, 1982) a locus for recruitment to a venom system. The recent sequencing of the genome of the venomous mammal *Solenodon paradoxus* has provided vivid evidence of the consequences of weaponization for a gene family which ancestrally undergoes evolution by birth-and-death (Casewell et al., 2019). Comparison of the *S. paradoxus* genome with that of other mammals revealed that birth-and-death was a widely occurring process, with the same locus within the kallikrein cluster showing evidence of it in the majority of the fifteen mammalian taxa investigated. Despite all lineages sharing 10 of the 15 KLK genes, there were numerous lineage-specific gain and loss events at this locus, as well as a smattering of “exonic debris”—the remnants of genes in the process of being expunged from the genome. In *S. paradoxus* however, which utilises kallikreins in its venom, there was an accumulation of copies that far outstripped that observed in any other taxon (Figure 4). This suggests that recruitment into venom creates a selection pressure for the accumulation of duplicate copies at a locus with an ancestral propensity for duplication.

## Reconstructing the Evolutionary History of a Multigene Family

### Phospholipase a2g2

The most comprehensive reconstruction of the evolutionary history of a toxin multigene family to-date was performed on the phospholipase a2 group 2 family, which is an important component of the venom of viperid snakes. To analyze a dataset comprising 110 genomic sequences from 93 species from across the animal kingdom, the study utilized a comparative approach combining manual genomic annotation, phylogenetics, selection rate estimates, and analysis of synteny. The following is a detailed discussion of the results of this study that pertain to the origins of a toxic function in venom for members of the gene family. Please refer to the original study (Koludarov et al., 2019) for additional details.

**FIGURE 5:**
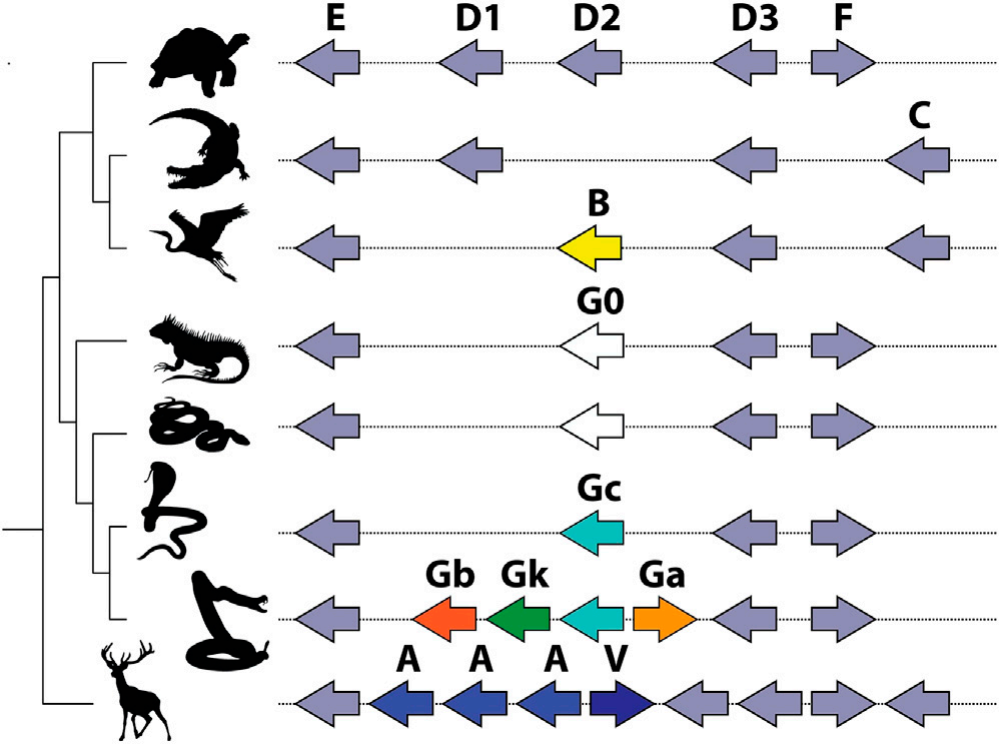
Schematic representation of Pla2g2 gene cluster in vertebrate lineages after (Koludarov et al., 2019). Note that all expansion within the cluster takes place at the same location, and all novel clades (g2B in birds; g2G in squamates; and g2A and g2V in mammals) are descended from the same subclade (g2D).

The Pla2g2 family is located in a remarkably syntenic genomic region, which facilitated the comparative approach of the study. All lineage-specific innovations within the cluster stem from the same locus, ancestrally associated with Pla2g2 subclade D (Figure 5). Mammals (Pla2g2A and g2V), birds (Pla2g2B), and squamate reptiles all exhibit lineage-specific derivations arising from within the D subclade. Subclade G of Pla2g2 is the lineage unique to squamate reptiles (lizards and snakes). The plesiomorphic form of this gene (given the name “Pla2g2G0”) was present in a single copy in the genomes of all squamates surveyed other than members of the Caenophidia (“advanced snakes”). In the caenophidian snakes (Elapidae, Viperidae and various non-front-fanged families formerly grouped in Colubridae), the gene is structurally derived (and given the name “Pla2g2Gc”). This gene (hereafter “g2Gc”) was present as a single copy in the genomes of all non-viperid caenophidians. In viperids, the G subclade has expanded considerably in association with its “recruitment” for a toxic function in venom and viperid genomes contain unique toxin-encoding forms such as “Gck” (a transitional form between Gc and Gk) “Gk”, “Ga”, and “Gb” (Dowell et al., 2016).

### Pla2g2 in Snake Venom

The MRCA of the advanced snakes may have been venomous (Jackson et al., 2017) and thus the potential exists at that early stage for positive selection acting upon genes encoding orally secreted toxins. It is unclear, however, whether g2Gc was in fact utilized as a venom toxin by early caenophidian snakes and whether this function may have provided the selection pressure leading to the fixation of this form in the (inferred) MRCA. However, the ancestral membrane-degrading activity of Pla2g2 gene products (Six and Dennis, 2000) exapts them for utilization as toxins or as antimicrobial components of innate immunity—note that these are not mutually exclusive as immune components are frequently co-opted for use as toxins (e.g., Whittington et al., 2008; Georgieva et al., 2011; Wong and Belov, 2012). Not all genes within the Gc group have been functionally characterized at this stage and data concerning their expression in various tissues is limited; these data are important in resolving the evolutionary pathways leading to the deployment of this gene family in the venom of viperid snakes. Regardless, Gck is selectively expressed in the venom gland of extant crotaline viperid snakes (Aird et al., 2017; Dowell et al., 2018). The homologous gene (Gc) is not expressed in the venom gland or accessory gland of the elapid snake O. hannah (despite being 94% similar in sequence to the viperid form), indicating that it is not utilized as a toxin by this species; it is also expressed at extremely low levels in pooled tissues, which may be indicative of its incipient toxicity (Vonk et al., 2013). While elapid snakes do utilize phospholipases as toxins, all known elapid Pla2 venom toxins are members of group 1, which is unrelated to group 2 (Fujimi et al., 2002).

### Toxic Function may Arise Prior to Duplication

**FIGURE 6:**
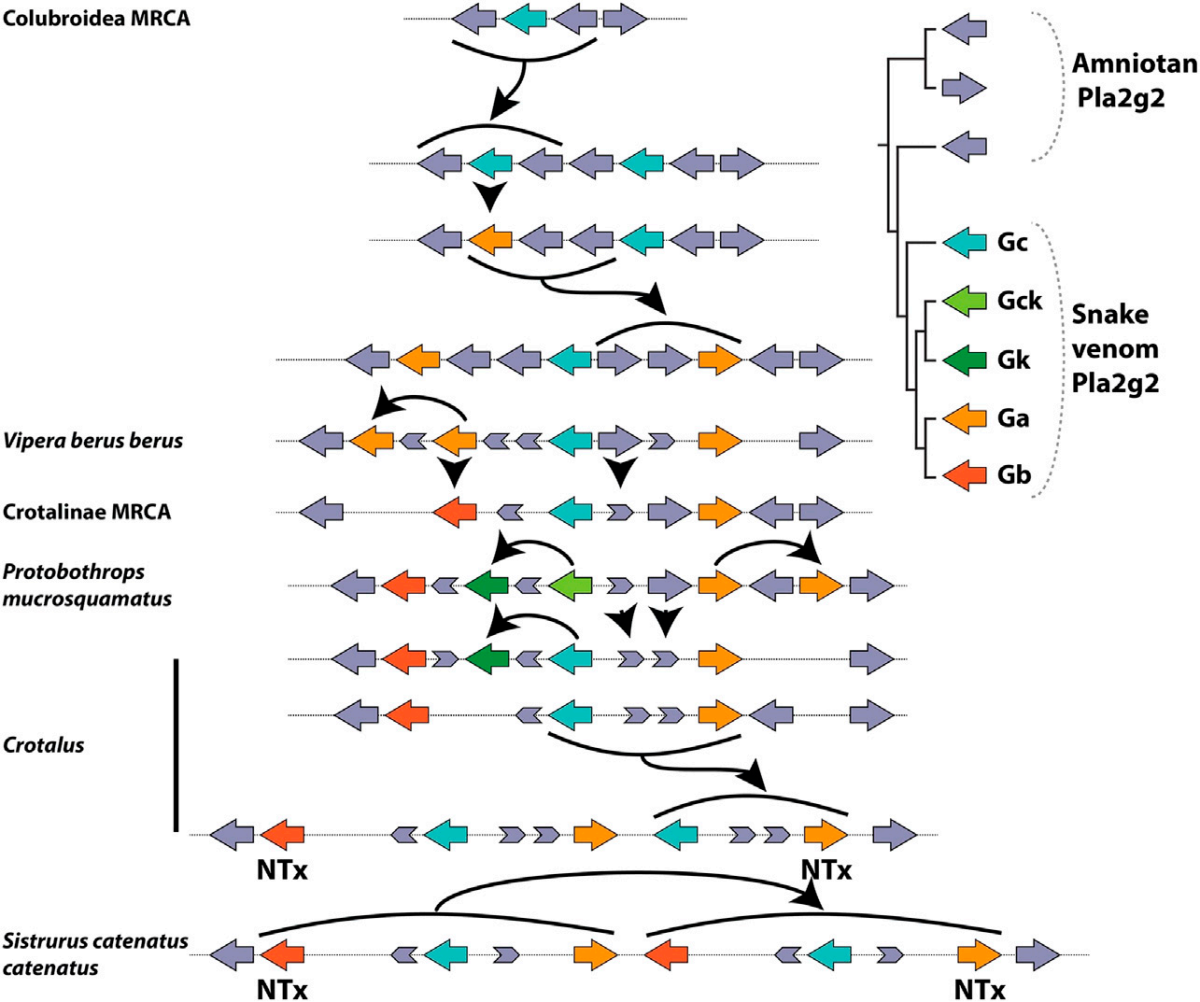
Lineage-specific expansion and diversification of the Pla2g2 subfamily in viperid snakes after (Koludarov et al., 2019). Note the presence of multiple fragments of “exonic debris” (mainly from g2E and g2D) that make possible the reconstruction of the evolutionary history, including all duplication events, of this genomic region in viperid snakes. Arrows indicate birth and death events. A number of the duplication events (bold arrows) do not involve single genes (unlike g2A of mammals) but rather small groups which are duplicated as units (“cassettes”), typically composed of a g2G gene flanked by parts of g2E and g2D.

In viperid snakes the plesiotypic form Gc, along with transitional and highly derived toxin forms, is expressed in the venom gland (Aird et al., 2017; Dowell et al., 2018). Thus, we infer that that the novel toxic function arose prior to duplication, possibly via co-option facilitated by a shift in tissue-specific expression patterns which resulted in its expression in the venom gland. Stochastic gene expression of this kind has been linked to the phenotypic diversity from which the origins of novel adaptations may arise (True and Carroll, 2002; Kaern et al., 2005; Woods, 2014). At present, our ability to pinpoint the origin of the toxin function in Viperidae is limited as we lack necessary transitional forms within that family. Duplication of the gene occurred sometime between the split of viperid snakes from the main stem of Colubroidea and the origin of the MRCA of extant Viperidae, which possessed additional copies. Thus, we infer that expansion at the locus occurred subsequent to the acquisition of the toxic function, similar to the pattern observed for *Solenodon paradoxus* (Figure 6).

The alternative possibility is that duplication occurred prior to “recruitment” to the venom system, giving rise to the new gene g2Ga. The protein encoded by this new gene, possessing by chance a greater toxicity than that of its parent gene (g2Gc), would have been selected for venom gland-specific expression and the parent gene was co-expressed due to the co-regulation of neighboring genes. This interpretation is complicated by the fact that g2Gc, which is initially a passively co-expressed (unselected) gene in the venom system according to this scenario, later evolves (in a Crotalinae-specific derivation) into the myotoxic g2Gk (a.k.a. “Lys49 Pla2s”) (Figure 6). Thus, instead of one, this alternate hypothesis requires as many as three “recruitment” events—one (of g2Ga) for the initial addition to the venom arsenal, a second one associated with the mutation of g2Gc into g2Gk and a third one in which g2Gb (which originates from a duplication of g2Ga which may pre- or post-date recruitment in this scenario) becoming a basic subunit of neurotoxic dimeric Pla2g2. In either case, changes in gene expression, which are untraceable at this level of analysis (and possibly lost to the sands of time) are crucially important in the initial acquisition of the novel, toxic function. Given the presence of additional “random” (unselected) steps in the latter scenario (duplication precedes novel function), we prefer the former (novel function precedes duplication—see below for a more detailed discussion). However, additional research is required to definitively differentiate between these hypotheses.

### The Role of Venom Delivery Systems in Recruitment

Viperid snakes diverged early from the main stem of the caenophidian lineage (which includes elapid snakes; the front-fanged lamprophiids *Atractaspis* and *Homoroselaps*; and many non-front-fanged venomous species) and the most striking synapomorphy of the family (Viperidae) is the possession of large, hollow fangs which are the sole tooth located on a mobile maxillary bone (Fry et al., 2012). These fangs, like those of other front-fanged snakes, are connected to the venom gland by an enclosed duct, and the gland itself is surrounded by compressor musculature which contracts during venom delivery. Together these anatomical components form a “high-pressure” venom delivery system, and viperids were the first lineage of snakes in which such a system evolved. That members of the Gc group of Pla2g2 apparently only became specialized for use as venom toxins after the divergence of Viperidae from other advanced snakes suggests that the acquisition of this function may have been associated with the evolution of a delivery system capable of inoculating the toxin directly into the muscle tissue of potential prey organisms.

This hypothesis is consistent with the subsequent diversification of the subfamily in viperid snakes, including the evolution of specialized myotoxic and presynaptically neurotoxic forms. Myotoxic Pla2 are likely to be more effective as toxins if delivered intramuscularly—a feat that non-front-fanged snakes, and even many front-fanged elapid snakes, are unlikely to be capable of. It should be noted, however, that myotoxic phospholipases have been independently recruited (from group 1 Pla2) as toxins in elapid snakes. These toxins are particularly enriched in the venoms of large species capable of exerting considerable bite force (e.g., *Pseudechis australis*—Georgieva et al., 2011) and in species which feed on prey items that lack a layer of subcutaneous fat (e.g., some hydrophiine sea snakes—Gopalakrishnakone et al., 1997; Phillips, 2002). It is plausible, therefore, that the ability to inoculate venom intramuscularly has played a role in the recruitment or enrichment of myotoxic Pla2g1 in elapid snakes—co-evolution of toxins and associated delivery anatomy is a reasonable expectation, and has been reported previously in toxicoferan reptiles (e.g.,—Fry et al., 2012) and cnidarians (Surm et al., 2019). As far as the present case of Pla2g2 in viperid snake evolution is concerned, additional investigation of snake bite force mechanics and feeding ecology is required to test these conjectures.

### Expansion of the Locus Following Recruitment

Subsequent to the acquisition of the toxic function, a series of duplication events expanded this lineage in viperid snakes, the first of which gave rise to two new isoforms—the g2Ga (acidic) and g2Gb (basic) venom Pla2s (Figure 6). These forms are more highly expressed in viper venom glands than the plesiotypic g2Gc form (Aird et al., 2017). Pla2g2G venom genes were duplicated in multiple lineages to produce genes that became subunits of heterodimeric neurotoxins in several *Crotalus* and *Sistrurus* species (French et al., 2004; Doley et al., 2010). As revealed by the arrangement and orientation of genes and exonic debris, these neurotoxins arose via independent duplications in each of these two genera (Figure 6; cf. Dowell et al., 2016), an example of convergent evolution made possible by the fact that a single point mutation is all that is required to “unlock cascading exaptations,” leading to the derivation of this potent toxin (Whittington et al., 2018). In parallel, g2Gc (the plesiotypic form) mutated (again in the absence of duplication) into g2Gck in Crotalinae (pit vipers), and an additional duplication of this form became the non-catalytic myotoxin (g2Gk) (Figure 6). In tandem with the evolution of these derived forms, the plesiotypical Gc and Gck appear to have had their expression suppressed, despite still being present in the genomes of many viperid snakes (Aird et al., 2017; Dowell et al., 2016; Dowell et al., 2018).

### An Ancestral Propensity for Duplication

The data described above concerning Pla2g2Gc suggest that duplication need not have been a prerequisite for the acquisition of a novel exophysiological function by this gene’s product in viperid snake venom. Although duplication may not have been proximally involved in the acquisition of the toxic function, the gene was part of a gene cluster, which is an important factor influencing its evolutionary trajectory and is consistent with the recruitment of many other toxins from multigene families (Fry et al., 2009). This is both because the locus clearly possesses an ancestral propensity for duplication, and because the gene’s function may have been shared with the sister genes (in case of the g2G ancestor, presumably g2D or even g2E/F/C). This redundancy probably decreased the evolutionary constraints on each gene. Importantly, all of the novel clades of Pla2g2 in distinct animal lineages originate from the same locus (ancestrally occupied by Pla2g2D—Koludarov et al., 2019), highlighting the influence of genomic context on duplication propensity and hence functional diversification (Jackson et al., 2019). It should be noted, however, that the original expansion events leading to the formation of the Pla2g2 cluster are ancient (>300 mya—Koludarov et al., 2019) and the general trend subsequent to this expansion appears to be toward reduction through gene loss, rather than further multiplication.

The pattern observed, in which both the emergence of novel functions and subsequent gene family expansion take place at the same locus in distantly related taxa, suggests that such loci have a deep ancestral propensity for mutation and duplication. The propensity for duplication is likely conferred by genomic structure, as particular arrangements of genetic material facilitate duplication (Reams and Roth, 2015). This propensity, however, may typically be constrained. The alternative, still advocated by some biologists (c.f. Dunn and Munro, 2016), is that duplications occur randomly throughout the genome and that regions only differ in copy number variation (CNV) due to the differential preservation of duplicates. This seems implausible for two reasons: 1) because exonic/intronic debris is typically evident following deletion (unless the deletions are extremely ancient events)—this debris was not detected throughout the genomes in the present study but only, *ex hypothesi*, in isolated regions in particular genomes (i.e., those in which birth-and-death is taking place); and 2) because down-regulation (“dosage sharing”) or silencing with methylation may facilitate the long-term preservation of segmental duplications in genomes despite the predictions of the dosage balance hypothesis (Assis and Bachtrog, 2015; Lan and Pritchard, 2016; Guschanski et al., 2017). Another alternative is that individuals in which deleterious duplications occur are strongly selected against and thus no evidence of these duplications persists in sequenced genomes, but this also seems an unnecessarily extreme speculation as it requires that such duplications be invariably lethal or render organisms sterile—i.e., individuals in which duplication takes place must produce no offspring. We note that it is far from novel to interpret patterns of duplication as non-random (e.g., Bailey et al., 2002), indeed we feel that this should be considered the null hypothesis in the absence of the evidence described above.

## Gene Duplication and the Evolution of Toxic Functions

### Recruitment in the Absence of Duplication

Several studies have downplayed the role of gene duplication in the acquisition of a toxic function by certain gene families in certain venomous lineages. In the platypus, for example, a majority of toxin-encoding genes do not exhibit lineage-specific expansions (Wong and Belov, 2012), rather toxins appear to have been recruited from gene families with pre-existing CNV, and no expansion has taken place subsequent to recruitment. In parasitoid wasps, duplication appears to have played even less of a role, with the predominant mode of venom diversification within and among lineages being shifts in cis-regulated gene expression in the absence of either gene duplication associated with acquisition of a toxin function, or gene deletion associated with its loss (Martinson et al., 2017).

### The Evolution of Novel Functions is Facilitated by Changes in a Gene Product’s Ecology

A protein’s function is fundamentally relational, i.e., defined interdependently as the consequence of interaction between one protein and another (Guttinger, 2018). We suggest that the emergence of novel functions becomes possible when a gene product’s context changes and it is exposed to a novel suite of interaction partners. This change of context is analogous to the changing ecology of an organism invading a new environment and the dynamic evolution that this may facilitate is therefore analogous to speciation via adaptive radiation (Jackson et al., 2019). A change of context may occur in multiple ways for a gene product, with or without gene duplication: following a stochastic change in expression pattern that sees a gene being expressed in a novel tissue, i.e., a tissue in which the gene product in question is not typically expressed (Kaern et al., 2005; Woods, 2014); following a structural change that modifies a protein’s interactive propensity (i.e., exposes it to a novel context in terms of potential partners for interaction); or following the evolution of a “delivery system” (e.g., long hollow fangs) capable of delivering the gene product into a novel context (e.g., muscle tissue of prey animals). Such changes of context may lead to the discovery of a “good trick” (Dennett, 1995) by fortuitously facilitating an interaction with a positive impact on fitness. It is at this point that a genuinely novel function emerges. If both functions (ancestral and derived) persist for the same gene, this may create pressure for duplication, as the multiple functions of the protein require segregation into discrete genes, a situation similar to that described in the “specialization,” “SF,” and “EAC” models (Hughes, 1994; Force et al., 1999; Innan and Kondrashov, 2010).

## Neofunctionalization and Recruitment, *Sensu Lato*

### Models and Pluralism

The precise sequence of events leading to the recruitment of Pla2g2Gc as a toxin cannot be definitively determined based on available data, however, it may superficially appear as though the single model the events described above most closely resemble is “SF” (Force et al., 1999). However, additional processes (e.g., “moonlighting” and “NF”) not described by that model also appear to have contributed to the origins of functional novelty within this gene family, and periods of “degeneration” (a feature of the SF hypothesis) may or may not have occurred (see below for further discussion). The “specialisation” model (Hughes, 1994) may describe the data even more closely, since recruitment as a toxin likely involved specialization for a novel function following an initially pleiotropic period. However, as with SF, this model does not describe a subsequent period of classic NF, which is observable within the viperid-specific toxin forms of Pla2g2. Perhaps then “IAD” (Näsvall et al., 2012) is the explanatory model that fits data most accurately. IAD, as described above, is a model that subsumes several other popular models into its narrative, and we suggest that any sufficiently fine-grained reconstruction of the evolutionary history of a gene family will require such a pluralistic approach. This raises the question of whether or not any single, relatively simplistic, model can ever adequately describe the emerge of novel functions.

Conant et al. (2014) suggested that a “pluralistic framework” incorporating multiple models may be the most appropriate way to understand the fate of duplicate genes and our analysis corroborates this assertion. The following paragraphs conjecturally describe events that may occur in episodes of “NF” (or “recruitment”). The term NF is used here to describe the emergence of novel functions at the molecular level, and not merely that emergence via Ohno’s model, and “recruitment” is used to mean the acquisition of a toxic function in venom, without or without duplication. This discussion should not be thought of as an attempt to define a new formal model, but rather to show how each of the previously proposed models may capture only part of the truth. Additional processes not described here likely occur in other cases.

### Innovation

The initial acquisition of a novel activity may occur: 1) when noisy expression patterns (Kaern et al., 2005; Woods, 2014) instigate a moonlighting scenario—a single copy gene fulfilling multiple functions by virtue of expression in multiple locations (Copley, 2014); or 2) when structural change facilitates interaction with novel partners, while maintaining the ancestral function. Note that in a moonlighting scenario there may be the acquisition of a “weak secondary function” (the “innovation” phase of IAD) in the absence of any structural change to the gene. The novel activity may then expose the gene to a distinct selection regime, which is the point at which activity becomes function. Selection may then facilitate the accumulation and fixation of further mutations. When a novel function is acquired by a single copy gene, this may create pressure for the creation of duplicate copies such that the multiple functions can be segregated between those copies, which may then specialize. Such a scenario, along with those in which duplication is positively selected due to the benefits of increased gene dosage or robustness (Innan and Kondrashov, 2010), may result in selection driving an increased duplication rate. An additional hypothesis describing positive selection on accumulation of duplicate genes suggests that this may occur when duplication results in the spontaneous origin of a novel “function,” however, this might be better referred to as a novel “propensity.” Again, it is debatable whether a trait qualifies as functional *prior* to making a contribution to organismal fitness (i.e., prior to selection) (Jackson and Fry, 2016; cf. ; Innan and Kondrashov, 2010).

### Amplification and Exochemical Escape

Certain novel functions lead to selection for increased expression of a gene product, which also contributes to the fixation of duplicate copies (Margres et al., 2017). This corresponds to the “amplification” phase of IAD, in which selection for the “weak secondary function” drives the accumulation of duplicates (Näsvall et al., 2012). Notably, in exochemical systems, since the interaction partners of gene products originate outside the body of the producing organism and the products are secreted extracellularly, the likelihood of a deleterious impact of mutations on fitness is decreased (allowing for their accumulation) and there are no (internal) stoichiometric constraints on dosage. Thus, products of duplicate genes in exochemical systems may escape both negative selection and down-regulation or silencing, thereby having the opportunity of diversifying and rapidly contributing to organismal fitness.

In contrast to the model proposed by Lan and Pritchard (2016) in which coregulation of tandem duplications delays sub- and neo-functionalization, this removal of constraint may facilitate rapid evolutionary divergence prior to genomic separation of duplicate genes. This phenomenon may be termed “exochemical escape,” where “escape” refers to the evasion of dosage balance constraints and thereby the solution to “Ohno’s dilemma” (Bergthorsson et al., 2007). A lack of dosage constraint on exochemical/extracellular proteins may also explain the lack of concordance between the evolution of these systems and the broader trend in conservation or deletion of duplicates following whole-genome duplications versus segmental duplications (Conant et al., 2014)—in exochemical systems, segmentally duplicated genes may persist even when they have many interaction partners and are involved in the formation of protein complexes.

### Diversification

Subsequent to initial duplication, specialization (a.k.a. “EAC”—Hughes, 1994; Innan and Kondrashov, 2010; also “diversification” in the IAD model) may occur, in which one copy of the gene maintains the original function and the other specializes for its exochemical role, e.g., a role in venom in viperid snakes. This specialization may result in selection for tissue-specific patterns of expression—although it has been suggested that the expression of tandem duplicates is likely to be co-regulated until one copy undergoes chromosomal displacement (Lan and Pritchard, 2016), available expression data clearly indicate that Pla2g2G are highly tissue-specific in their expression and that neighboring genes (Pla2g2E and Pla2g2D) are not expressed in the venom gland (Vonk et al., 2013; Dowell et al., 2016; Aird et al., 2017).

### More is Better

This specialization may lead to increased selection on dosage, driving the accumulation of duplicate genes now specifically expressed within the exochemical system (further “amplification,” following “diversification”). This is particularly likely for systems in which more gene product is “better,” either leading to a more toxic venom (Margres et al., 2017) or more effective response to infection (e.g., mammalian g2A). At this point, classic Ohno-style redundancy occurs, as multiple gene copies represent both a larger target for mutational change (and thus a network for exploring phenotype space) and each becomes less constrained by purifying selection (Aird et al., 2017). This in turn leads to NF, in Ohno’s sense of the term, in which specific gene copies evolve interactions with novel partners.

### Co-Option Leads to Neofunctionalization

The aforementioned sequence describes a sequence (and a hypothesis in need of further testing) that loosely subsumes moonlighting, specialization/SF and NF into a single temporal series. It is similar to IAD but includes additional rounds of amplification following specialization for an exochemical role. As with the first round of amplification, the accumulation of duplicates facilitates classic NF via redundancy. Models in which duplication is central to the evolution of functional novelty have dominated discussion in recent years, but the co-option of single copy genes is likely also widespread (True and Carroll, 2002; Martinson et al., 2017) and may be the first step on the pathway toward “NF.” Assertions that functional novelty may often precede duplication are nothing new. Indeed, they date back at least to the work of Serebrovsky (1938, referenced in Taylor and Raes, 2004), who discussed the pleiotropic effects of a single gene being distributed between daughter genes following duplication (see also Piatigorsky and Wistow, 1991). More recently, Hughes explicitly states that a period of gene sharing precedes duplication-facilitated specialization (Hughes, 1994). Whether these models, or that which we have outlined in the previous paragraph, should be considered “SF” (Force et al., 1999) is perhaps a moot point. The formal SF model includes “degeneration” (of regulatory elements or functional structures) following duplication. While this may occur, the significant consequence of duplication, particularly in terms of venom toxins, appears to be “EAC” (Hughes, 1994; Des Marais and Rausher, 2008), which in turn leads to NF proper (Ohno, 1970). This pattern conforms with the analyses of Assis and Bachtrog (Assis and Bachtrog, 2015), who demonstrated that SF was rare in comparison to conservation, specialization, or NF, and indicated that SF may be merely a stage in the evolutionary series leading toward NF.

### Models are Maps, not Territory

In any case, formal models are rarely more than schematics, and there is little reason to expect real world sequences of events to conform to them precisely. Thus, while we do not believe we have reconstructed a history that conforms to rigorously defined “SF,” clearly that history resembles this model, just as it resembles elements of several others. Hargreaves et al. (Hargreaves et al., 2014) previously argued that venom toxins likely acquire their toxic functions via SF rather than NF. In this they were making a point of difference with much of the molecular evolutionary work done in the field of toxinology (e.g., Reza et al., 2005; Lynch, 2007; Fry et al., 2012), in which it had been previously well accepted that Ohno-style NF was the dominant process of protein “weaponization.” Indeed, as more research is conducted on the genomes of venomous organisms, it is becoming increasingly evident that even for toxin evolution there can be no one size fits all explanation. For example, in the king cobra genome, evidence of moonlighting was reported for some toxin genes alongside considerable evidence of toxin-specific gene family expansion, which appeared to confirm the classic NF model’s applicability to toxin evolution (Vonk et al., 2013). A similar pattern of gene family expansion was observed in the genome of the anemone *Actinia tenebrosa* (Surm et al., 2019) and has been observed in a huge number of studies of various venom taxa, a comprehensive review of which is beyond the scope of the present article. The platypus genome, on the other hand, revealed a pattern in which toxin genes are recruited from families with ancestral CNV, and no evidence of lineage-specific (i.e., associated with the toxin function) expansion was uncovered for most of these families (Wong and Belov, 2012). In parasitoid wasps, yet another pattern was observed in which duplication appears to play almost no role; rather, acquisition and loss of toxic function was facilitated by changes in cis-regulated gene expression (Martinson et al., 2017).

### Neofunctionalization = “Origin of a Novel Function”

In our study, as described above, we have detected a pattern that suggests that both co-option facilitated by changes in gene expression and lineage-specific gene family expansion are important in toxin evolution. We therefore agree (with Hargreaves et al., 2014 and others) that Ohno’s model does not account for all the details, but feel that it describes an important part of the process characteristic of certain venom toxin families, namely the expansion of these families via duplication and the attendant positively selected evolution of multiple novel functions. We further recommend that the term “NF” not be too narrowly defined, as it, etymologically, merely refers to the origin of novel functions. Ohno’s initial coinage was a catchy one and we would like the usage of this term to be legitimate, despite the fact that in its narrow definition is does not capture all the details. Those that have read Ohno’s monumental publication of 1970 (Ohno, 1970), know that his thought was expansive and that he described processes akin to SF working alongside the NF for which he is remembered. In this sense he was like Darwin, whose thoughts on evolution extended beyond Natural Selection and the conceptual tools of what became, in the 20th Century, Neo-Darwinism. Thus “Darwinism” is more expansive than “Neo-Darwinism” and “NF” may be legitimately considered more expansive than its formal definition suggests.

This “highway to NF” that we conjecture has shaped the evolution of certain branches of the Pla2g2 family may be unique to rapidly evolving exochemical systems or may be more widespread. In other cases of multiplication within the Pla2g2 family, however, diversification takes place much more sedately. This is evidenced by the fact that plesiotypical D-clade proteins in turtles and alligators are more similar to each other and even to EFC-clade proteins than they are to the divergent forms of mammals, birds or squamates (Koludarov et al., 2019). Thus, sequence divergence and the antiquity of the duplication event are not tightly correlated in this gene family—the functional role of the gene in question dictates the dynamism of its evolution.

### Functions Exist at the Organismal Level

At this point it is necessary to reiterate a fact often overlooked by studies investigating the origins of novel functions at the molecular level—functions exist at the organismal level. There is an implicit assumption (perhaps transmitted from physics) that causal pathways must flow from small things like genes “upwards” to large things like organisms. Such an assumption has no place in evolutionary biology, in which selection pressures which originate at the level of organisms interacting with their environment shape the evolution of lineages. In biology, a “function” is the purpose of trait that justifies its existence via its contribution to the fitness of the organism that possesses it (Jackson and Fry, 2016). In order for a gene product to acquire a novel function as a venom toxin, there must be a confluence of factors – an appropriate activity, an appropriate site and level of expression, and a delivery mechanism capable of inoculating the toxin. Note that the requirements of the delivery system are dictated by the activity and available concentration of the toxin—venom systems are integrated, and the evolutionary dynamics of their components reflects this complexity. In the case of Pla2g2G, the “recruited” gene occurs at a locus with an ancestral propensity for duplication. This is likely the case for many toxins, as being part of a multifunctional multigene family presents obvious advantages for both the derivation of new activities in general, and new toxic activities specifically. Thus, such multigene families are exapted for recruitment into venom systems. However, the evidence garnered from the reconstruction of this gene family’s evolutionary history indicates that the duplication rate at that locus was dramatically elevated *subsequent to the acquisition of the toxic function*. The same pattern was observed for kallikrein genes in the genome of *Solenodon paradoxus* (Casewell et al., 2019). Thus, these studies provide a nice example of the kind of “downwards causation” that is likely ubiquitous in evolutionary biology (Noble, 2013; Ellis, 2015; Noble et al., 2019), in which the state and behavior of the organism as a whole influence the state of its constituent molecules just as much as the opposite.

## Conclusion

There are many models of the evolution of novel functions at the molecular level and each of them may describe a possible process that occurs in nature. It is unlikely, however, that any of them captures the full range of possible pathways through which novelty emerges, or even tells the full story of any particular pathway. This is to be expected—at their best, models are akin to accurate maps, and maps are always coarse-grained representations of the realities they describe. In this article we have reviewed a number of these models and data that has been interpreted as evidence of their involvement in the acquisition, by proteins, of toxic functions in venom. We prefer to call the acquisition of such a function “recruitment,” regardless of the specific pathway(s) involved, because this term captures the fact that a toxin is a “weaponized molecule.”

This broad usage of the term “recruitment” should be understood as distinct from its narrower usage to describe a hypothesis in which the origin of a toxic function occurs subsequent to “duplication of a bodily protein.” This process may occur, but it is certainly not the only way in which a toxic function emerges. Neither is expression of a potentially toxic molecule in an oral gland (or any secretory tissue associated with a venom system) sufficient for recruitment to occur. It is likely that the oral glands of all vertebrates secrete a plethora of molecules (e.g., enzymes involved in pre-digestion, as well as antimicrobial peptides) that could potentially be deployed as venom toxins. Despite this, the majority of vertebrates are clearly non-venomous. This is because a venom toxin is a component of an integrated system which includes a delivery mechanism and which serves an ecological function. In the absence of this ecological function—active delivery of the secretion to a target organism to facilitate feeding, defense or (in this case of “venomous” parasites) surreptitious feeding—the potential toxicity of many secretory molecules remains untapped.

While there has been debate about whether “recruitment” or “restriction” (NF or SF) is the primary route through which the evolution of a toxic function occurs (Hargreaves et al., 2014), this debate misses the aforementioned point—neither of these processes is sufficient unto itself for the acquisition of such a function. Indeed, both of them are likely involved in recruitment (*sensu lato*), either in separate cases or as distinct stages within a single process. Certainly, there can be no doubt that NF as described by Ohno (1970) is an active process in toxin multigene families, in which redundancy conferred by the accumulation of duplicates in tandem arrays facilitates the origin of novel activities. These novel activities may become novel functions as attacking new targets within the physiology of an envenomed organism is an important process that contributes to the evolutionary success of venoms (Casewell et al., 2019). However, NF (or birth-and-death) within a toxin multigene family is not evidence that the same process was involved in the initial acquisition of a toxic function in venom by members of the family. In any case, the redundancy among models of gene evolution—several of which are either the same or subsume one-another—suggests that arguing over which is the “primary” model involved in toxin evolution may be unproductive.

As well as recommending an expansive definition for “recruitment,” we suggest that “NF” is a suitable term for the origin of novel molecular functions in general. In this, we argue simply that the etymology is appropriate, and that simpler terminology is often preferable to a proliferation of models with increasingly elaborate acronyms that only confuse the issue. Genomic data, particularly when combined with expression data, now present an extremely rich source of information about molecular evolution. When a comparative approach is employed, these data facilitate the reconstruction of evolutionary histories at an unprecedented level of detail. As evolutionary toxinology marches further into the genomic era, we expect further evidence that myriad variations on a theme exist in nature, and that each case, when reconstructed to a sufficiently fine-grained degree, is unique unto itself. In evolution whatever can happen, will happen.

## Data Availability Statement

The data analyzed in this study is subject to the following licenses/restrictions: Data can be made available on request. Requests to access these datasets should be directed to jcoludar@gmail.com.

## Author Contributions

TJ conceived and wrote the bulk of the manuscript. IK contributing conception, writing and editing of the manuscript and made the figures.

## FUNDING

IK was funded by the German Research Foundation (DFG), grant RE3454/6-1. TJ was supported by a grant from the National Health and Medical Research Council (NHMRC) to the Australian Venom Research Unit.

## Conflict of Interest

The authors declare that the research was conducted in the absence of any commercial or financial relationships that could be construed as a potential conflict of interest.
